# Clinical outcomes of mild to moderate coronavirus disease 2019 patients treated with Regdanvimab in delta-variant outbreak: Retrospective cohort study

**DOI:** 10.1097/MD.0000000000035987

**Published:** 2023-11-10

**Authors:** Hyeong-Jun Noh, Jin Hwa Song, Sin Young Ham, Yeonkyung Park, Ha-Kyeong Won, Soo Jung Kim, Keun Bum Chung, Choon Kwan Kim, Young Mee Ahn, Byoung-Jun Lee, Hye-Rin Kang

**Affiliations:** a Division of Pulmonary and Allergy Medicine, Department of Internal Medicine, Veterans Health Service Medical Center, Seoul, Republic of Korea; b Division of Infectious Disease, Department of Internal Medicine, Veterans Health Service Medical Center, Seoul, Republic of Korea; c Division of Pulmonary, Allergy and Critical Care Medicine, Department of Internal Medicine, Hallym University Kangnam Sacred Heart Hospital, Seoul, Republic of Korea; d Department of Internal Medicine, Graduate School of Medicine, Seoul National University, Seoul, Republic of Korea.

**Keywords:** antibodies, COVID-19, delta variants, monoclonal, Regdanvimab, SARS-CoV-2

## Abstract

Regdanvimab is a novel neutralizing antibody agent used for the treatment of coronavirus disease 2019 (COVID-19). However, the effectiveness of regdanvimab in delta-variant patients has rarely been investigated. We examined the clinical outcomes and adverse events in COVID 19 patients treated with regdanvimab in the delta-variant era. Data were collected from laboratory-confirmed COVID-19 hospitalized patients who received regdanvimab in 2021 and categorized into pre-delta and delta variant groups. The primary outcome was the need for oxygen therapy. Rescue therapy, clinical improvement, and adverse events were analyzed. Among 101 patients treated with regdanvimab, 31 (30.7%) were delta patients and 49 (48.5) were pre-delta patients. 64.4% were male, the mean age was 60.3 years, and 70 patients (69%) had at least one underlying disease. The median interval from symptom onset to injection was 4 days. Twenty-three patients (23%) needed oxygen therapy, including 9 (29%) in the delta and 8 (16.3%) in the pre-delta group. (*P* = .176) The risk of early oxygen supplement was higher in the delta group (adjusted hazard ratio (aHR), 6.75; 95% confidence interval(CI), 1.53–29.8). The in-hospital survival rate was 100%, and no patients were admitted to the intensive care unit. Adverse events occurred in 43% of patients:13 (42%) delta patients and 23 (47%) pre-delta patients had any adverse events (*P* = .661). Patients treated with regdanvimab 4 days after symptom onset showed a favorable prognosis (aHR, 0.26; 95% CI, 0.26–0.91). We found that the high-risk mild to moderate COVID-19 patients treated with regdanvimab showed similar disease progression in delta-variant patients and pre-delta variants; however, we need to be more closely observed delta-variant patients than those in the pre-delta group despite regdanvimab treatment due to rapid disease aggravation.

## 1. Introduction

The coronavirus disease 2019 (COVID-19) pandemic has become a global health problem^[1]^ While most patients show non-severe disease, some progress to severe disease.^[2,3]^ Although vaccination protects patients against the severe acute respiratory syndrome coronavirus 2 (SARS-CoV-2) virus, treatment medications remain an important strategy.^[4]^ The use of monoclonal antibodies (mABs) has been investigated as a potential therapy for preventing disease progression.^[5,6]^

Regdanvimab is an mAB that targets the receptor-binding domain of the viral spike protein and has strong neutralizing activity against SARS-CoV-2.^[7]^ In a phase II/III study, regdanvimab reduced the requirement for hospitalization or oxygen therapy,^[8]^ and in February 2021, the Food and Drug Administration of Korea approved the use of regdanvimab for patients with mild SARS-CoV-2 infection. It has also been approved in Europe, Indonesia, Brazil, Peru, and Australia.^[9]^

Several retrospective studies have demonstrated the efficacy of regdanumab.^[10–12]^ However, the antiviral effect of regdanvimab on the SARS-CoV-2 delta variant (B.1617.2) has been demonstrated only in vivo and in vitro. There is a lack of clinical data on the use of regdanvimab for patients with the delta variant.^[13]^

Therefore, we aimed to review treatment outcomes and adverse events in COVID-19 patients treated with regdanvimab, as well as the outcome differences between patients with the delta variant and those with wild type or other pre-delta variants.

## 2. Methods

### 2.1. Study design and participants

This retrospective cohort study included hospitalized adults with COVID-19 who were administered regdanvimab between February 10, 2021, and December 31, 2021. This study was conducted at an infectious disease hospital for COVID-19 in Korea. The eligibility criteria for regdanvimab treatment were patients with real-time reverse transcriptase polymerase chain reaction-confirmed COVID-19, mild COVID-19 disease status^[14]^ with any COVID symptoms, room-air peripheral capillary oxygen saturation ≥ 94%, patients not requiring oxygen supplementation, the onset of symptoms no more than 7 days previously, and at least one risk factor for the development of severe illness: (1) patients ≥ 50 years old; (2) patients with at least one medical condition (cardiovascular disease, chronic lung diseases, diabetes, kidney disease, chronic liver disease, or obesity), and (3) patients with pneumonia.^[15]^ Asymptomatic patients or those requiring oxygen therapy were not eligible for regdanvimab treatment Regdanvimab was administered after obtaining written informed consent from the patient. The dose of regdanvimab was 40 mg/kg per single dose and was administered by intravenous infusion over 60–90 minutes, with full monitoring of vital signs.. According to Korean guidelines,^[16]^ all patients received treatment according to the standard of care for COVID-19. Patients with COVID-19 symptoms with oxygen saturation of > 94% in room air and those who did not require supplemental oxygen were classified as having mild to moderate disease. Patients who required low-flow oxygen were classified as having severe disease status, and those who required ICU care or high-flow oxygen were classified as having a critical disease status.^[17]^

### 2.2. Classification of SARS-CoV-2 variants

**Figure 1.**
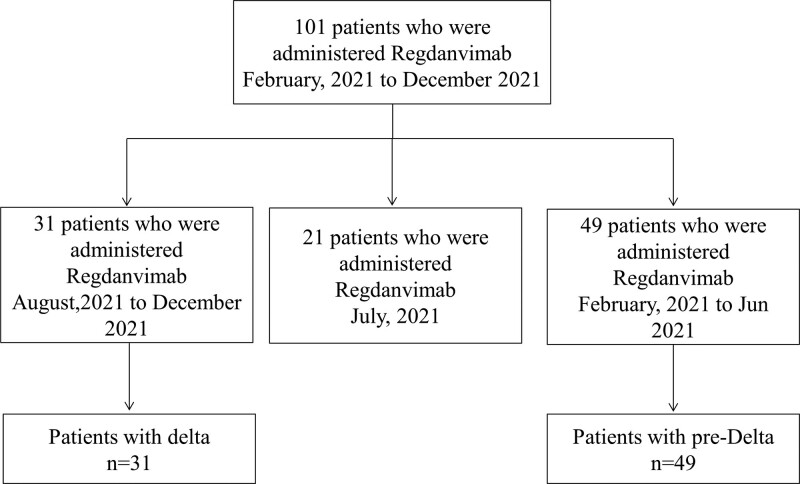
Flow chart of patients’ enrollment and grouping.

According to data from the Korea Disease Control and Prevention Agency, until July 2021, most SARS-CoV-2 was wild-type, and 20% was the alpha (B.1.1.7) variant. The delta variant of SARS-CoV-2 accounted for < 20% of cases from July 2021, whereas > 90% of patients infected with SARS-CoV-2 were shown to have a delta variant in August 2021.^[18,19]^ We adapted the definition of the delta variant in Korea according to a study by Rye et al and divided patients into 2 categories: pre-delta and delta variants.^[18]^ Patients diagnosed with COVID-19 between February 2021 and June 2021 (the period when the delta variant detection rate was < 10%) were classified into the pre-delta group, and those diagnosed between August 2021 and December 2021 (the period when the delta variant detection rate was > 90%) were assigned to the delta group. Patients diagnosed with COVID-19 in July 2021 were excluded from these 2 group analyses (Fig. 1).

### 2.3. Baseline and follow-up data collection

Age, sex, height, body weight, underlying comorbidities, initial COVID-19 symptoms, viral load (from the cycle threshold value of the reverse transcriptase-polymerase chain reaction), diagnosis date, radiologic evidence of pneumonia, regdanvimab infusion date, symptoms, vital signs, and laboratory results were collected from medical records. Data collected for rescue therapy for COVID-19 included details on the use of remdesivir, steroids, antibiotics, or oxygen supplements. Any adverse events after regdanvimab administration were collected from medical records. Data on laboratory abnormalities (leukopenia, increased alanine aminotransferase, and inflammation markers) after regdanvimab treatment were collected from patients who underwent a follow-up blood test.

### 2.4. Clinical outcomes

The primary outcome was the proportion of patients who required supplemental oxygen therapy. The secondary outcomes for disease aggravation were the proportion of patients requiring rescue treatment (remdesivir, dexamethasone, or antibiotics) and the time taken to require rescue therapy (remdesivir, dexamethasone, or antibiotics), as well as the proportion of patients with pneumonia aggravation, intensive care unit transfer, and in-hospital mortality. The secondary endpoints for disease improvement included time to clinical recovery, time to fever subside in 3 successive days without antipyretics, and time to National Early Warning Score 2 (NEWS2) of 0. Data on adverse events were collected from the time of drug infusion until the patients’ discharge.

### 2.5. Statistical analysis

The baseline characteristics and laboratory results of COVID-19 patients were represented as means with standard deviations or medians with interquartile ranges. Underlying diseases or patients’ initial symptoms were described as proportions. The clinical outcomes are presented as numbers and proportions. The median time from regdanvimab treatment to the requirement for rescue therapy was analyzed in patients who required rescue therapy. COVID-19 aggravation was defined as the need for oxygen supplementation during the treatment period, and the odds ratio of COVID-19 aggravation was estimated using binary logistic regression analyses. Improvement of COVID-19 symptoms was calculated as the median interval from the onset of symptoms to NEWS2, resolution of symptoms, and fever subsiding on 3 successive days without antipyretics. Kaplan–Meier curves and log-rank tests were used to analyze time-dependent outcomes, and the adjusted hazard ratio (aHR) with a 95% confidence interval (CI) was estimated using Cox regression for the delta and pre-delta groups. Adverse events are represented as numbers and proportions. Differences between patients in the delta and pre-delta groups were compared using the chi-square test or Fisher exact test for categorical variables and analysis of variance for continuous variables. Statistical significance was set at *P* < .05. All analyses were performed using Stata 17.0 (Stata Corp, College Station, Texas, USA).

### 2.6. Ethics statement

This study was approved by the Institutional Review Board of the Veteran Health Service Medical Center (IRB no. BOHUN 2022-02-017) and the requirement for informed consent was waived.

## 3. Results

### 3.1. Baseline characteristics of patients

One hundred and one patients received regdanvimab treatment between February 10 and December 31, 2021. Among them, 31 patients were classified into the delta group and 49 were classified into the pre-delta group according to the diagnosis date (Fig. 1).

The baseline characteristics, initial clinical manifestations, and radiographic and laboratory findings of the patients are presented in Table 1. The mean age of the population was 60.3 ± 13.8 years, and 69.3% of patients had at least one underlying disease, including hypertension, heart disease, diabetes mellitus, chronic lung diseases, chronic liver diseases, chronic renal failure, and cancer. The patients in the delta group showed a significantly lower body mass index than those in the pre-delta group (mean 24.2 and 26.3, respectively; *P* = .017). The proportion of patients with initial pneumonia on radiological imaging was 61%. The delta group had a significantly higher proportion of patients with pneumonia than the pre-delta group (*P* = .046), and patients in the delta group had a higher proportion of patients with initial dyspnea (*P* = .003). The median interval from symptom onset to injection was 4 days. Patients in the delta group had a significantly shorter interval between symptoms and injections than those in the pre-delta group (*P* = .009).

**Table 1:** Baseline characteristics and initial clinical manifestation of the study patients.

Characteristics	All patients (n = 101)	Delta patients (n = 31)	Pre-delta patients (n = 49)	*P* value
Age, yr	60.3 ± 13.8	60.5 ± 17.8	63.0 ± 10.4	.424
Sex, Male no. (%)	65 (64.4)	19 (61.3)	37 (75.5)	.176
BMI (kg/m^2^)	25.2 ± 3.7	24.2 ± 3.3	26.3 ± 3.9	.017
≥30 kg/m^2^	11 (10.9)	1 (3.23)	8 (16.3)	.071
Underlying disease, no (%)				
Any of the listed conditions	70 (69.3)	25 (80.7)	35 (71.4)	.354
Hypertension	34 (33.7)	14 (45.2)	18 (36.7)	.454
Heart disease	26 (26.0)	12 (38.7)	13 (26.5)	.395
Diabetes mellitus	28 (27.7)	11 (35.5)	19 (33.9)	.120
Chronic lung disease	9 (8.9)	5 (16.1)	4 (8.2)	.272
Chronic renal failure	1 (0.99)	0 (0.0)	1 (2.0)	1.000
Cancer	10 (9.9)	5 (16.1)	4 (8.2)	.272
Chronic liver disease	3 (3.0)	1 (3.2)	2 (4.1)	1.000
Viral load (Ct value), mean ± SD	18.9 ± 6.9	18.6 ± 7.8	19.4 ± 6.5	.6167
Initial saturation, median (IQR)	97 (96–98)	97 (96–98)	97 (96–98)	
Presence of initial pneumonia on chest X-ray, No (%)	62 (61.4)	21 (67.7)	22 (44.9)	.046
Initial symptoms, no (%)				
Headache	20 (19.8)	8 (25.8)	8 (16.3)	.392*
Fever & chilling sense	40 (39.6)	10 (32.3)	18 (36.7)	.683
Dyspne	9 (8.9)	7 (22.6)	1 (2.0)	.003*
Cough	67 (66.3)	22 (71.0)	30 (61.2)	.373
Myalgia	49 (48.5)	49 (48.5)	25 (44.6)	.385
GI symptoms	10 (9.9)	3 (9.7)	4 (8.2)	.815*
Laboratory findings				
White cell count, median (IQR) per mm^3^	4460 (3750–6000)	4540 (3980–6450)	4400 (3910–5650)	.094
high-sensitivity C-reactive protein ≥ 10 mg/L	57 (56.4)	17 (54.8)	24 (49.0)	.610
Lactate dehydrogenase ≥ 250 U/L	39 (38.6)	13 (41.9)	16 (32.7)	.400
Creatinine, mg/dL	0.9 ± 0.3	0.9 ± 0.4	0.9 ± 0.2	.407
Alanine aminotransferase > 40 U/L	17 (16.8)	4 (12.9)	9 (18.4)	.519
Aspartate aminotransferase > 40 U/L	17 (16.8)	4 (12.9)	6 (12.2)	.931
D-dimer, mg/L	0.5 ± 0.4	0.7 ± 0.6	0.4 ± 0.3	.012
Median days from symptom onset to medication, median (IQR)	4 (3–6)	3 (2–5)	4 (3–6)	.009

BMI = body mass index, Ct = cycle threshold, GI = gastrointestinal, IQR = interquartile range, No. = number, SD = standard deviation.

Data are presented as mean ± SD or median with IQR.

* Fisher exact test.

### 3.2. Clinical outcomes and rescue therapy

Table 2 presents the results of this study. Among the 101 patients, 23 (22.8%) required oxygen therapy, 38 (37.6%) showed radiological pneumonia aggravation, 18 (17.8%) required additional antiviral therapy, 26 (25.7%) required systemic steroids to treat COVID-19, and 8 (7.9%) required systemic antibiotics to treat combined bacterial pneumonia. None of the patients was admitted to the intensive care unit or died prior to discharge. The median interval from symptom onset to symptom resolution was 10 days, and the median interval to fever subsidence in 3 successive days without antipyretics was 9 days. The median interval from the onset of symptoms to the NEWS2 score of 0 was 7 days.

**Table 2:** Outcomes and additional treatment.

Outcomes	All patients (n = 101)	Groups according to SARS-CoV-2 variant (n = 80)		*P* value
		Delta patients (n = 31)	Pre-delta patients (n = 49)	
**Primary endpoint**				
Requiring oxygen supply*, no. (%)	23 (22.8)	9 (29.0)	8 (16.3)	.176
**Secondary endpoint for disease aggravation**				
Aggravation of pneumonia†, no. (%)	38 (37.6)	11 (35.5)	17 (34.7)	.942
Requiring additional rescue therapies, no. (%)				
Anti-viral agent (Remdesivir)	18 (17.8)	8 (25.8)	5 (10.2)	.065
Systemic steroids	26 (25.7)	9 (29.0)	5 (10.2)	.031
Systemic antibiotics	8 (7.9)	2 (6.5)	6 (12.2)	.400
Median time from Regdanvimab, days (IQR)				
To suppling oxygen	2 (1–3)	1 (1–2)	3 (2–3)	.177
To Remdesivir	2 (1–3)	1.5 (1–4)	2 (2–4)	.502
To steroid	1 (0–2)	1 (1–2)	2 (2–4)	.240
To antibiotics	1.5 (1–6)	11 (2–20)	1 (0–2)	.127
Admission to intensive care unit, no. (%)	0 (0.0)	0 (0.0)	0 (0.0)	N/A
Death, no. (%)	0 (0.0)	0 (0.0)	0 (0.0)	N/A
Median hospital stay, days (IQR)	9 (8–11)	8 (7–10)	10 (9–12)	.025
**Secondary endpoints for disease improvement**				
Median interval from onset of symptoms, days (IQR)				
To resolution of symptoms	10 (8–13)	10 (7–13)	9 (8–13)	.992
To fever subside in 3 consecutive days without antipyretics	9 (6–10)	7.5 (6–10)	9 (6–10)	.591
To National Early Warning Score 2 of 0	7 (4–10)	6 (4–11)	6 (4–10)	.866

* All patients who received oxygen were supplied with a low-flow oxygen supply system.

† Pneumonia aggravation measured according to follow-up chest radiography and clinical course.

SARS-CoV-2 = severe acute respiratory syndrome coronavirus 2.

**Figure 2.**
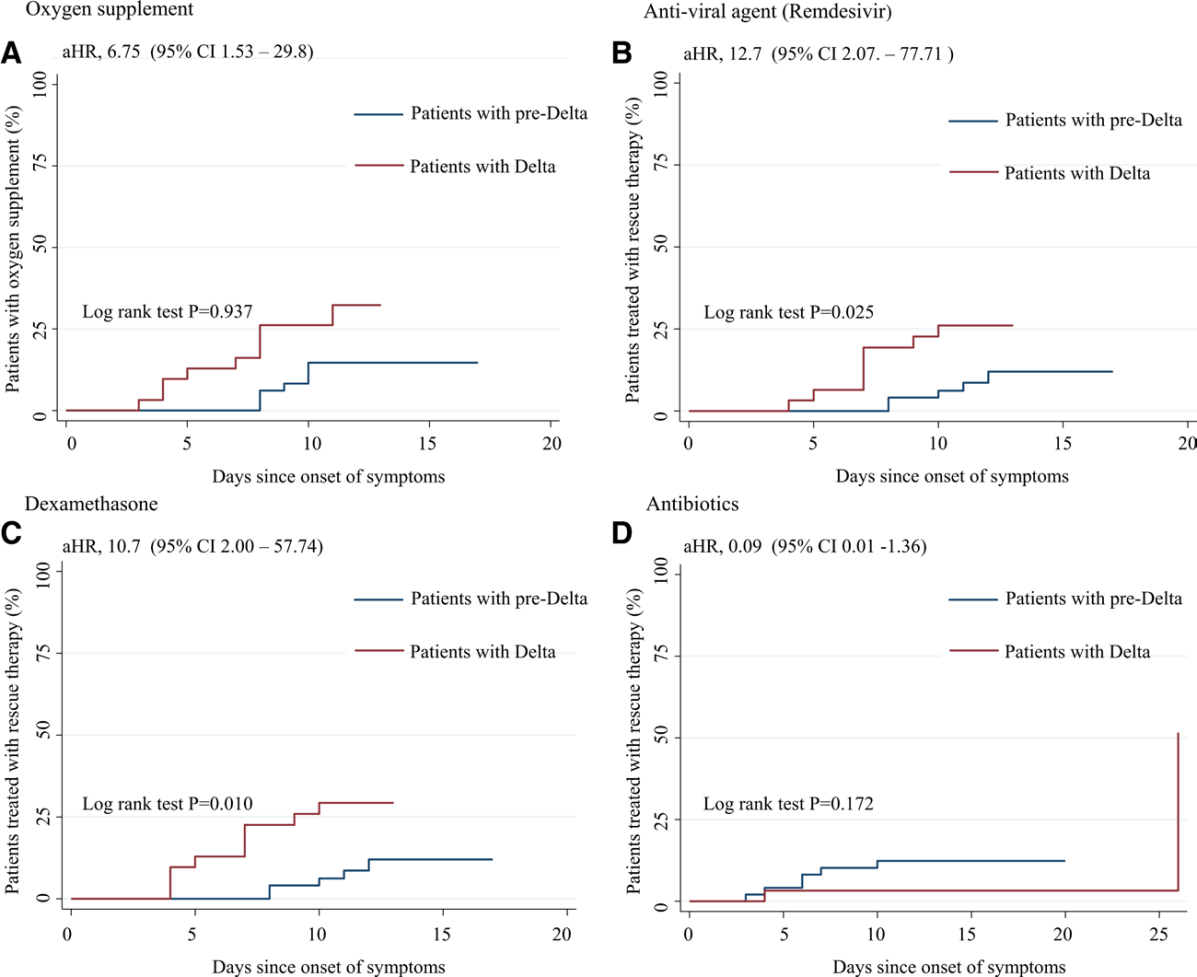
Time to rescue therapy between patients with delta and patients with pre-delta. (A) Oxygen supplement, (B) anti-viral agent (remdesivir), (C) dexamethasone, and (D) antibiotics. aHR was performed for delta patients compared with pre-delta patients adjusted for age, sex, body mass index, delta variant, initial pneumonia, C-reactive protein, D-dimer, aspartate aminotransferase, lactate dehydrogenase, SARS-CoV-2 viral load, and interval between onset of symptoms and injection of regdanvimab. aHR = adjusted hazard ratio, SARS-CoV-2 = severe acute respiratory syndrome coronavirus 2.

**Figure 3.**
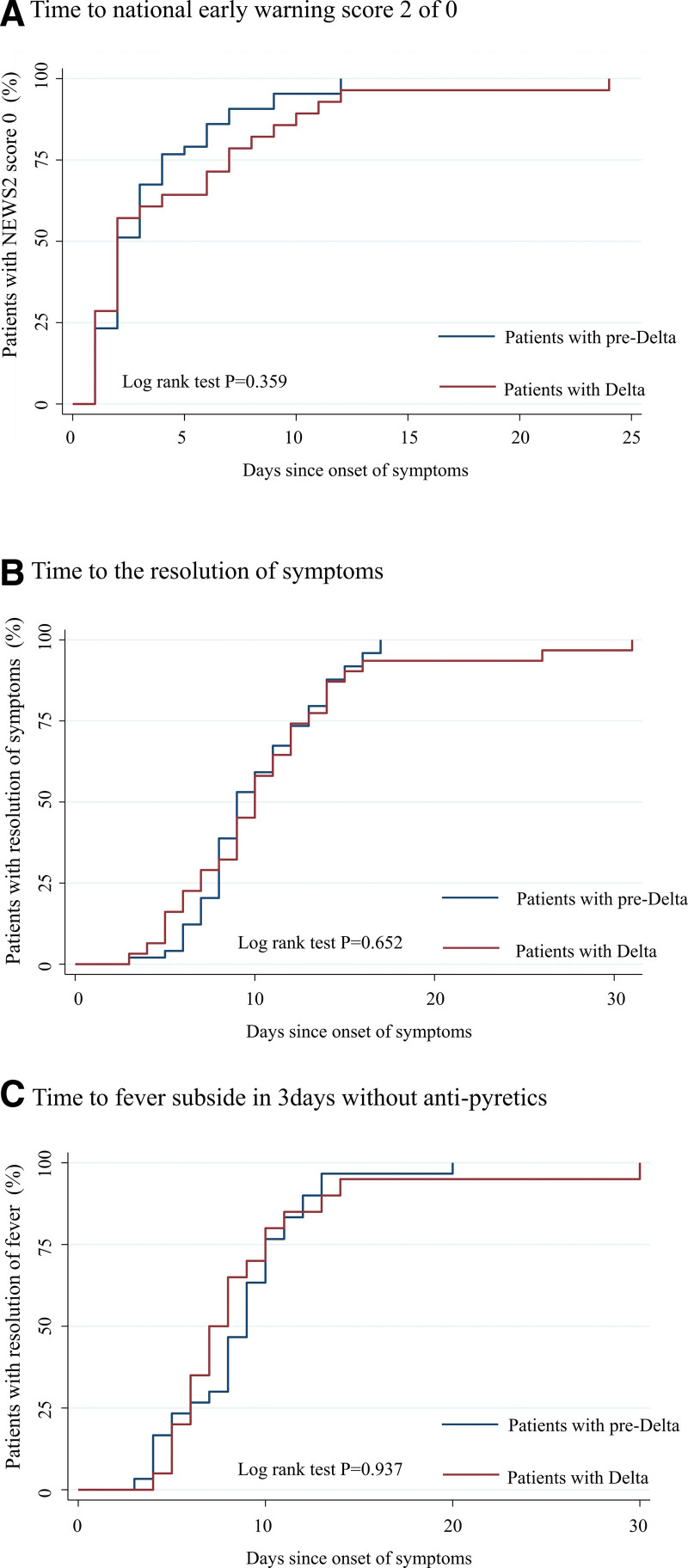
Clinical outcome difference between patients with delta and patients with pre-delta variants. (A) Time to National Early Warning Score 2 of 0. (B) Time to resolution of symptoms. (C) Time to fever subsidence in 3 consecutive days without antipyrectics.

In the prespecified subgroup analyses, 9 patients (29%) in the delta group and 8 patients (16%) in the pre-delta group required oxygen therapy, indicating no significant difference between the 2 groups (*P* = .176). Kaplan–Meier analysis revealed that patients in the delta group tended to have a higher risk of early oxygen supplementation than those in the pre-delta group and showed a higher hazard ratio (aHR, 6.75; 95% CI, 1.53–29.8) (Fig. 2A). More patients in the delta group required steroid therapy than those in the pre-delta group in rescue therapy (29% vs 10%, *P* = .031) (Table 2). Moreover, the interval of administered rescue therapy of remdesivir and dexamethasone was shorter in patients in the delta than those in the pre-delta group (aHR, 12.7; 95% CI, 2.07–77.71 for delta and aHR, 10.7; 95% CI, 2.00–57.7 for pre-delta) (Figs. 2B and C). There were no differences in the use of antibiotics between the 2 groups (delta group aHR, 0.09; 95% CI 0.01–1.36) (Table 2, Fig. 2D). There were no significant differences between the 2 groups regarding disease improvement indicators. The median interval from onset to symptom resolution was 10 days in the delta group and 9 days in the pre-delta group (*P* = .992). The interval for fever to subside on 3 successive days without antipyretics was 7.5 days in patients in the delta group and 9 days in the pre-delta group (*P* = .591), and the interval to NEWS2 of 0 was 6 days in the 2 groups (*P* = .866) (Table 2). Both groups showed comparable time intervals with a median recovery interval from onset to resolution of symptoms, fever subside, and NEWS2 of 0 (Fig. 3). The median hospital stay was shorter in the delta group than in the pre-delta group (median 8 vs 10 days, *P* = .025).

### 3.3. Factors associated with disease aggravation in patients treated with Regdanvimab

**Figure 4.**
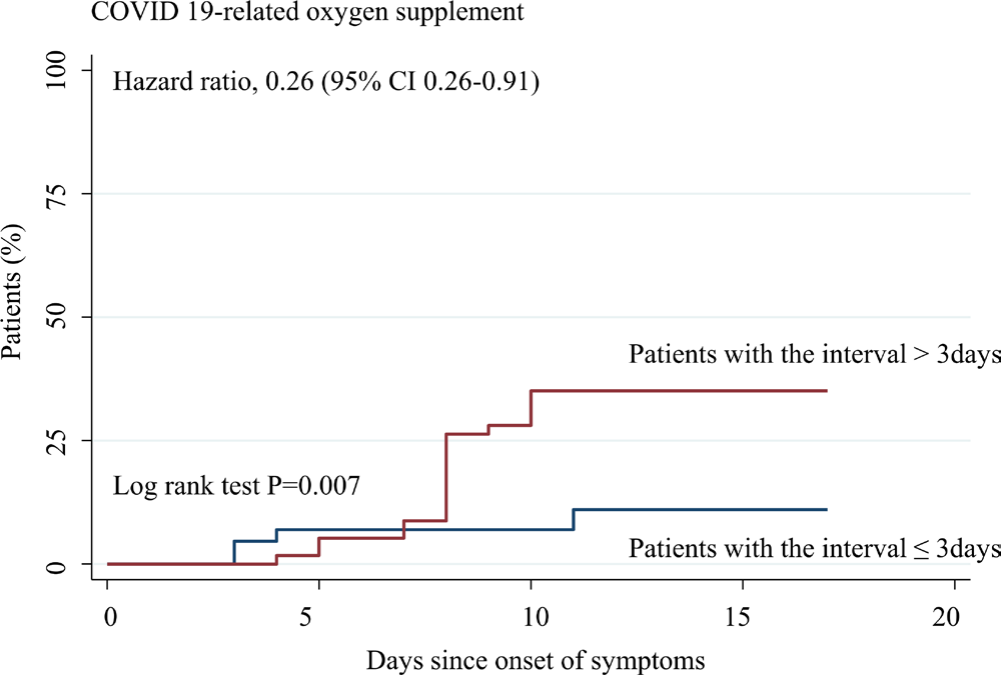
Clinical outcome difference according to interval of symptoms to injection of Regdanvimab. aHR was performed for patients with an interval of < 4 days compared with those with an interval ≥ 4 days adjusted for age, sex, body mass index, delta variant, initial pneumonia, C-reactive protein, d-dimer, aspartate aminotransferase, lactate dehydrogenase, SARS-CoV-2 viral load, and interval between symptom onset and the injection of regdanvimab. aHR = adjusted hazard ratio, SARS-CoV-2 = severe acute respiratory syndrome coronavirus 2.

Multivariate logistic regression analysis of factors associated with disease progression showed that higher levels of high-sensitivity C-reactive protein were associated with disease aggravation (adjusted odds ratio, 1.11; 95% CI, 1.01–1.22). In addition, the interval from symptom onset to medication injection date of < 4 days was inversely associated with disease aggravation (adjusted odds ratio, 0.24; 95% CI, 0.08–0.70). However, age, sex, body mass index, and initial pneumonia did not affect the disease outcomes among patients treated with regdanvimab (Table 3, Fig. 4).

**Table 3:** Factors associated with COVID-19 aggravation in patients treated with Regdanvimab.

	Univariate analysis	*P*-value	Multivariate analysis	*P* value
	OR (95% CI)		OR (95% CI)	
Age, yr	1.01 (0.97–1.04)	0.703	1.04 (0.93–1.17)	.486
Sex, male	0.98 (0.38–2.51)	0.966	1.31 (0.14–12.16)	.486
BMI, kg/m^2^	1.02 (0.91–1.16)	0.714	1.25 (0.78–12.16)	.349
Delta variant	2.45 (0.81–7.48)	0.114	12.8 (0.71–233.75)	.085
Cardiovascular disease	1.15 (0.41–3.17)	0.792		
Diabetes mellitus	1.32 (0.49–3.52)	0.582		
Any cancer	0.74 (0.15–3.73)	0.715		
Viral load, Ct value	0.95 (0.88–1.01)	0.153	0.82 (0.67–1.01)	.056
Vaccination	0.77 (0.25–2.32)	0.639		
Initial pneumonia on image	10.91 (2.40–49.55)	0.002	0.99 (0.03–33.4)	.997
Leukocytosis	1.15 (0.93–1.42)	0.194		
High-sensitivity C-reactive protein, mg/L	1.06 (1.03–1.08)	<0.001	1.11 (1.01–1.22)	.028
Lactate dehydrogenase, U/L	1.01 (1.00–1.02)	0.002	1.00 (0.99–1.02)	.632
Creatinine, mg/dL	2.81 (0.53–15.07)	0.227		
Aspartate aminotransferase, 40 U/L	1.03 (1.00–1.06)	0.041	0.94 (0.82–1.07)	.339
D-dimer, per mg/L	3.03 (1.08–8.47)	0.035	35.13 (0.33–3791.54)	.136
PT (s)	1.00 (0.97–1.03)	0.991		
Interval from onset of symptoms to injection day ≤ 3 days	0.24 (0.08–0.70)	0.009	0.01 (0.01–0.45)	.020

BMI = body mass index, Ct = cycle threshold, OR = odds ratio, PT = prothrombin time.

* Adjusted for age, sex, BMI, delta variant, viral load, pneumonia, high-sensitivity CRT, lactate dehydrogenase, aspartate aminotransferase, d-dimer, and interval from onset of symptoms to injection day.

### 3.4. Adverse events

The adverse events are described in Table 4. A total of 46 patients (46%) experienced at least one adverse event, and adverse drug reactions were considered in 30 patients (30%). The most frequently reported adverse event was fever, followed by hypertension during regdanvimab administration, headaches, and newly developed skin lesions. The most common laboratory abnormalities after the medication were abnormal liver function tests and elevated inflammatory markers (13 out of 40 patients underwent follow-up blood tests). No fatal adverse events were reported.

**Table 4:** Adverse events.

Adverse event	All patients (n = 101)	Delta patients (n = 31)	Pre-delta patients (n = 49)	*P* value
Any adverse event, no (%)	46 (45.5)	13 (41.9)	23 (46.9)	.661
Adverse drug reaction, no (%)	30 (29.7)	7 (22.6)	16 (32.7)	.332
Adverse events, no (%)				
Hypertension*	12 (11.9)	1 (3.2)	11 (22.45)	.019
Fever	22 (21.8)	7 (22.6)	10 (20.4)	.817
Headache	5 (5.0)	2 (6.5)	2 (4.1)	.636
Skin lesion	3 (3.0)	1 (3.2)	2 (4.1)	.844
Chill	2 (4.3)	0 (0.0)	2 (4.1)	.255
Myalgia	2 (2.0)	0 (0.0)	2 (4.1)	.255
Hypotension	2 (2.0)	0 (0.0)	0 (0.0)	NA
Others†	4 (4.0)	1 (3.23)	2 (4.08)	.844
Laboratory abnormality, no. (%)	n = 40	n = 10	n = 21	
Leukopenia	3/40 (7.5)	0/10 (0.0)	3/21 (14.3)	.209
Increased ALT	13/40 (32.5)	3/10 (30.0)	5/21 (23.8)	.713
Elevated inflammatory marker	13/40 (32.5)	2/10 (20)	10/21 (47.6)	.140

ALT, alanine aminotransferase.

* Hypertension was defined as high blood pressure that developed during drug administration. Systolic blood pressure increased by ≥20 mm Hg at baseline and increased by >150 mm Hg in absolute terms.

† One patient complained of chest tightness, and the other complained of feeling tired and hot in their nose.

The analysis of patients with delta and pre-delta variants showed comparable frequencies of adverse events and adverse drug reactions (*P* = .661 and *P* = .332, respectively) (Table 4).

## 4. Discussion

The present study examined the clinical outcomes of patients with mild to moderate COVID-19 treated with regdanvimab, who were analyzed according to delta variants. Among the study population, 23% of patients showed disease aggravation and required additional oxygen therapy, even after treatment with regdanvimab. Although patients in the delta group showed no statistically different ratio of oxygen supplements compared to the pre-delta group, they showed a higher risk of rapid oxygen requirement and rescue treatments (dexamethasone or remdesivir). There was no significant difference in adverse events between the delta- and pre-delta patients.

Although regdanvimab was shown to have therapeutic potential in in vitro experiments,^[13]^ its effectiveness has not been demonstrated in patients with delta-variant COVID-19. Our study showed the experience of regdanvimab in delta variants of COVID 19 patients. In our population, regdanvimab showed no difference in efficacy in the primary outcome between the delta variant group and the pre-delta variant group, but the delta patients showed more rapid disease progression than pre-delta patients. These results suggest that the efficacy of regdanvimab in patients with delta variants may not be the same as that in patients with pre-delta variants.

Compared to the patients in the previous study, the delta variant patients in this study showed a significant proportion of disease progression. The proportion of patients who required oxygen therapy was 29.0% in the delta group. These findings are higher than the 4.0% reported in the phase II/III clinical trial of regdanvimab and 8.1% in a study by Lee et al^[8,10,15]^ In the present study, the median time to symptoms was ten days in the delta variant group, which is also relatively more prolonged than the 5.35 days reported in the phase II/III clinical trial of regdanvimab.^[8,10,15]^

In the present study, 45.5% of patients experienced adverse events and 29.7% experienced adverse drug reactions, comparable to the adverse event rate (28.3%) reported in a previous II/III clinical trial. All adverse events were mild, and no fatal adverse events occurred. mAB therapy may be associated with anaphylaxis during infusion^[20]^ however, there were no anaphylactic events. Surprisingly, we found that 11.9% of the patients had infusion-related hypertension. Hypertension was controlled quickly and without intervention after the infusion.

Injection of regdanvimab 4 days after symptom onset promptly decreased the risk of aggravation. This suggests that a rapid decision to treat patients with regdanvimab is critical to patient outcomes. Furthermore, the initial viral load was not related to poor outcomes, which is consistent with the findings of previous studies.^[21]^

These findings have several clinical implications. First, our results were analyzed according to the virus variant era, and our data suggest that we expect different clinical outcomes according to the virus variant. Treatment of COVID-19 patients with any medication may show different clinical results according to the virus variant, in terms of the delta and new variants. The present study findings reinforce previous findings that we need to be aware of viral variants. Second, our study identified a risk factor for poor outcomes in patients treated with regdanvimab. This medication is effective when administered within 4 days of symptom onset.

The strengths of the present study are as follows. First, our study showed various clinical outcomes. Disease aggravation was analyzed by assessing the requirement for oxygen, other rescue therapy needs, radiological aggravation of pneumonia, intensive care unit admission, and in-hospital death. Disease improvement was analyzed by assessing the resolution of symptoms, fever subsiding in 3 successive days without antipyretics, and NEWS2 of 0. Analyzing various clinical outcomes helped assess patient outcomes in detail. Second, these results show the real-world clinical outcomes of COVID-19 patients during the pandemic era. The present study was conducted in a secondary hospital dedicated to infectious diseases and included patients within the general population, not those with rare diseases. Therefore, our results may reflect patient outcomes in a real-world setting compared with tertiary hospital or clinical trial data.

The present study had some limitations. First, our study did not include a follow-up viral load after regdanvimab treatment. This was a retrospective study conducted in the usual practice, and a follow-up reverse transcriptase polymerase chain reaction assay was not recommended to measure the viral load. Second, the study had a comparably short follow-up period, and data were collected only during the administration period. Long-term outcomes after discharge from the hospital were not collected or analyzed.

In conclusion, our study showed the clinical outcomes of regdanvimab-treated patients compared to those of patients with delta and pre-delta variants. The proportion of patients with aggravated disease was similar between those with delta variants and those with pre-delta variants. However, patients with the delta variant showed a higher risk of rapid aggravation than those with the pre-delta variants.

## Author contributions

**Conceptualization:** Hyeong-Jun Noh, Jin Hwa Song, Sin Young Ham, Yeonkyung Park, Ha-Kyeong Won, Soo Jung Kim, Keun Bum Chung, Choon Kwan Kim, Young Mee Ahn, Byoung-Jun Lee, Hye-Rin Kang.

**Data curation:** Hyeong-Jun Noh, Jin Hwa Song, Sin Young Ham, Yeonkyung Park, Ha-Kyeong Won, Soo Jung Kim, Keun Bum Chung, Choon Kwan Kim, Young Mee Ahn, Byoung-Jun Lee, Hye-Rin Kang.

**Formal analysis:** Hyeong-Jun Noh, Jin Hwa Song, Byoung-Jun Lee, Hye-Rin Kang.

**Investigation:** Jin Hwa Song, Yeonkyung Park, Ha-Kyeong Won, Keun Bum Chung, Choon Kwan Kim, Byoung-Jun Lee.

**Software:** Hyeong-Jun Noh, Hye-Rin Kang.

**Supervision:** Sin Young Ham, Yeonkyung Park, Ha-Kyeong Won, Soo Jung Kim, Byoung-Jun Lee, Hye-Rin Kang.

**Validation:** Young Mee Ahn.

**Visualization:** Hyeong-Jun Noh, Hye-Rin Kang.

**Writing – original draft:** Hyeong-Jun Noh, Ha-Kyeong Won, Young Mee Ahn, Hye-Rin Kang.

**Writing – review & editing:** Jin Hwa Song, Sin Young Ham, Yeonkyung Park, Ha-Kyeong Won, Soo Jung Kim, Keun Bum Chung, Choon Kwan Kim, Byoung-Jun Lee, Hye-Rin Kang.
